# Post-stroke patients with moderate function have the greatest risk of falls: a National Cohort Study

**DOI:** 10.1186/s12877-019-1377-7

**Published:** 2019-12-26

**Authors:** Wycliffe E. Wei, Deirdre A. De Silva, Hui Meng Chang, Jiali Yao, David B. Matchar, Sherry H. Y. Young, Siew Ju See, Gek Hsiang Lim, Ting Hway Wong, Narayanaswamy Venketasubramanian

**Affiliations:** 1 Health Services Research Unit, Singapore General Hospital, Level 4, 226 Outram Road, Singapore, 169039 Singapore; 2 Department of Neurology (Singapore General Hospital Campus), National Neuroscience Institute, Singapore General Hospital, Outram Road, Singapore, 169608 Singapore; 3 Duke-NUS Medical School, Singapore, 8 College Road, Singapore, 169857 Singapore; 4 Saw Swee Hock School of Public Health, National University of Singapore, 12 Science Drive 2, #10-01, Singapore, 117549 Singapore; 5 Health Services & Systems Research, Duke-National University of Singapore Medical School, Singapore, 8 College Road, Singapore, 169857 Singapore; 6 Center for Clinical Health Policy, Duke University Medical Center, Durham, North Carolina USA; 7 Duke University Medical Center, Durham, North Carolina 27710 USA; 8 Department of Rehabilitation Medicine, Changi General Hospital, 2 Simei Street 3, Singapore, 529889 Singapore; 9 Health Promotion Board, 3 Second Hospital Avenue, Singapore, 168937 Singapore; 10 Department of General Surgery, Singapore General Hospital, General Hospital, Outram Road, Singapore, 169608 Singapore; 11 Raffles Neuroscience Centre, Raffles Hospital, Level 9, 585 North Bridge Road, Singapore, 188770 Singapore

**Keywords:** Stroke, Falls, Function, Modified Rankin scale

## Abstract

**Background:**

Stroke patients have increased risks of falls. We examined national registry data to evaluate the association between post-stroke functional level and the risk of low falls among post-stroke patients.

**Methods:**

This retrospective cohort study analyzed data from national registries to examine the risk factors for post-stroke falls. Data for patients who suffered ischemic strokes and survived the index hospital admission was obtained from the Singapore National Stroke Registry and matched to the National Trauma Registry, from 2011 to 2015. The primary outcome measure was a low fall (fall height ≤ 0.5 m). Competing risk analysis was performed to examine the association between functional level (by modified Rankin score [mRS] at discharge) and the risk of subsequent low falls.

**Results:**

In all, 2255 patients who suffered ischemic strokes had recorded mRS. The mean age was 66.6 years and 58.5% were men. By the end of 2015, 54 (2.39%) had a low fall while 93 (4.12%) died.

After adjusting for potential confounders, mRS was associated with fall risk with an inverted U-shaped relationship. Compared to patients with a score of zero, the sub-distribution hazard ratio (SHR) increased to a maximum of 3.42 (95%CI:1.21–9.65, *p* = 0.020) for patients with a score of 2. The SHR then declined to 2.45 (95%CI:0.85–7.12, *p* = 0.098), 2.86 (95%CI:0.95–8.61, *p* = 0.062) and 1.93 (95%CI:0.44–8.52, *p* = 0.38) for patients with scores of 3, 4 and 5 respectively.

**Conclusions:**

An inverted U-shaped relationship between functional status and fall risk was observed. This is consistent with the complex interplay between decreasing mobility (hence decreased opportunity to fall) and increasing susceptibility to falls. Fall prevention intervention could be targeted accordingly.

(263 words)

## Background

Falls are common after strokes, with stroke survivors having an estimated 14% risk of falling in the first month [1]. Besides injuries, those who fall experience activity limitation, increased dependence, and fear of falling [2]. These form barriers to social and community participation, and negatively impact quality of life [3]. Overcoming these are challenging, as patients require significant cognitive and emotional adjustment to successfully adopt coping strategies [4]. It is thus important to develop effective interventions to reduce risks of a post-stroke fall.

Seven major risk factors for falls among community stroke survivors have been identified and in descending order of risk are: impaired mobility, reduced balance, use of psychotropic medications, disability in self-care, depression, cognitive impairment and a history of falls [5]. Several of these are components of functional status which may be a more significant predictor of post-stroke falls. One measure of functional status is the modified Rankin Scale (mRS), which is easily implemented and used in both clinical and stroke trials [6].

Nevertheless, observational studies on the risks of post-stroke falls are limited. Existing studies examining the risks of post-stroke falls in the community are mostly small and cross-sectional in design [7, 8]. Large prospective studies are few. In a systematic review that identified 16 prospective observational studies, half had sample sizes below 100 and only two had a sample size above a thousand [5]. Overall, the evidence may not paint the full picture of the risk factors of falls and stroke, and data on a broad spectrum of stroke patients is needed. Only then can targeted strategies be put in place to reduce the risk of falls and its consequences on the wider stroke population.

Falls captured in trauma registries differ from typical methods used in other observational studies that use self-reports through diary or interview. Trauma registries capture more severe falls that result in presentations for medical care with detailed outcome measures such as the severity and site of injury [9, 10]. In contrast, studies relying on self-reported outcomes pick up minor and non-injurious falls. For example, a survey of community dwelling elderly showed that 67% of falls resulted in injuries, with 8.6% reporting head injuries [11]. Severity of injuries usually cannot be determined in these studies. The use of trauma registries in the study of falls thus provides an additional perspective and information on more severe falls that result in healthcare utilization.

### Aim and hypothesis

This study aims to use nation-wide registry data to evaluate how functional status, as represented by the mRS, is associated with post-stroke falls. We hypothesize that lower mRS, representing milder functional deficits, is associated with reduced risks of fall.

## Methods

This retrospective cohort study analyzed data from national registries to examine the risk factors for post-stroke falls. Singaporean patients who survived the index admission for stroke were identified from the National Stroke Registry (NSR). The records were then matched to the National Trauma Registry (NTR) by national identification number and de-identified prior to analysis. For both registries, data between the years 2011 to 2015 was used.

NSR, established in 2002, collects information on stroke cases diagnosed in all public hospitals. NSR is notified of stroke cases through medical claims made to the Singapore Ministry of Health, hospital inpatient discharge summaries and the national death registry. Information collected by the registry (e.g. risk factors and functional outcome scores) is then obtained from medical records and uploaded to the registry in standardised electronic forms [12].

NTR, established in 2011, captures cases that present to emergency departments of all public hospitals with a diagnosis relating to trauma. Information collected include injury severity and outcome measures. Some fields are captured from electronic records automatically while others are collected by data coordinators from the records [10].

The registries are updated with death outcomes by referencing the national death registry. The inclusion criteria, data collection, data cleaning and data quality audit processes of the registries have been described in previous studies [13, 14]. Only ischemic strokes were included in the main analysis.

### Outcome of interest

The primary outcome was a low fall as captured by the NTR, which is defined by same-level falls or those with fall heights of up to 0.5 m [10]. The height of fall recorded in the NTR was determined from patient histories based on reference heights (e.g. furniture heights) [10]. Only low-falls were considered as, based on the literature, they are more likely to be related to frailty in contrast to trauma from other injury mechanisms (road traffic injuries, falls from height – e.g. ladders or high-rise buildings in urban areas, machinery injury or other blunt mechanisms of injury) which likely have different risk factors and may introduce unnecessary heterogeneity [10, 15, 16]. Event dates in both national registries were matched prior to de-identification, and only post-stroke falls were considered.

### Independent variables

Demographic and clinical variables from the NSR, as well as injury characteristics from the NTR for patients with falls, were used in the analyses. Injury severity was described by the Abbreviated Injury Score (AIS), where a score of three and above represents serious injury. Variables that have been associated with frailty, falls or stroke were included in the analysis. Antiplatelet treatment and anticoagulation at discharge were also included in the model. Post-stroke functional level was measured by the final recorded mRS during the index admission for stroke. Patients without a completed Rankin score were excluded from the main analysis.

### Statistical analysis

Bivariate analysis was performed by using the *t*-test for continuous variables, and the *χ*^2^-test or Fisher’s Exact test was used for count data. Competing risks analysis, with a low fall as the event of interest and death as the competing event, was performed to evaluate associations with the risk of falls. Data analysis was performed using STATA version 13.0 (Stata Corp, College Station, Tx, USA) and the level of significance was set at *p* < 0.05.

### Sensitivity analyses

Sensitivity analysis was performed by extending the competing risks analysis for falls to all strokes in the NSR from 2011 to 2015. Cox Proportional Hazards regression for post-fall survival was also performed on all strokes who suffered low falls from 2011 to 2015.

### Ethics approval

The second last author’s Institutional Review Board granted ethical approval for this retrospective study, as required prior to gaining access to data from the NSR and the NTR, which is de-identified prior to release for research, password-protected and access limited to the premises of the National Registry of Diseases Office (NRDO). Consent was not obtained because information was anonymized and de-identified prior to analysis as per the NRDO protocol.

## Results

**Fig. 1:**
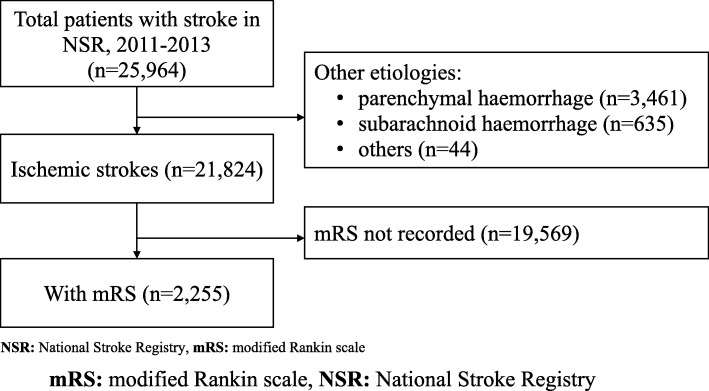
Study Flow Diagram of Main Analysis. **NSR:** National Stroke Registry, **mRS:** modified Rankin scale

Of 25,946 patients that entered the NSR between 2011 to 2013, 21,824 (84.1%) suffered strokes of ischemic aetiology, followed by parenchymal haemorrhage (13.3%; 3461 patients) and subarachnoid haemorrhage (2.5%; 635 patients). Among ischemic strokes, 2255 (10.3%) patients had recorded Rankin scores (Fig. 1 and Table 1).

**Table 1:** Characteristics of Ischemic Stroke Patients in Singapore from 2011 to 2015, With and Without Recorded Modified Rankin Scores

Variables		With Rankin Score (*n* = 2255)		Without Rankin Score (*n* = 19,569)		*p*-value
		Frequency	Proportion	Frequency	Proportion	
Age (mean, sd)		66.6	(12.8)	67.7	(13.3)	< 0.001
Blood Sugar Level, mmol/L (mean, sd)		8.9	(4.47)	9.2	(4.67)	0.014
Haemoglobin at Admission, g/dL (mean, sd)		13.8	(1.89)	13.5	(2.11)	< 0.001
Gender						0.910
Male		1319	58.5%	11,472	58.6%	
Female		936	41.5%	8097	41.4%	
Ethnicity						0.436
Chinese		1690	74.9%	14,858	75.9%	
Malay		389	17.3%	3120	15.9%	
Indian		146	6.5%	1331	6.8%	
Others		30	1.3%	260	1.3%	
Smoking						0.005
Non-smoker		1321	58.6%	11,224	57.4%	
Ex-smoker		281	12.5%	2800	14.3%	
Current-smoker		628	27.8%	4893	25.0%	
Diabetes Mellitus		950	42.1%	8515	43.5%	0.209
Hypertension		1838	81.5%	16,122	82.4%	0.301
Hyperlipidaemia		2057	91.2%	17,668	90.4%	0.154
Atrial Fibrillation		458	20.3%	3874	19.8%	0.562
History of TIA or Stroke		324	14.4%	3963	20.3%	< 0.001
Anti-platelet at discharge		1920	85.1%	16,214	82.9%	0.006
Anti-coagulant at discharge		266	11.8%	1623	8.3%	< 0.001
Discharge Home		1563	69.3%	11,090	56.7%	< 0.001
On Stroke Pathway		2110	93.6%	14,327	73.2%	< 0.001
Final Modified Rankin Score (*n* = 2324)						
0		450	20.0%			
1		519	23.0%			
2		379	16.8%			
3		357	15.8%			
4		381	16.9%			
5		169	7.5%			
Deaths by December 2015		93	4.1%	4051	20.7%	< 0.001
Sustained a Fall before Death		0	0.0%	349	1.8%	< 0.001
No Fall before Death		93	4.1%	3702	18.9%	< 0.001
Low Falls, Total Number		54	2.4%	1996	10.2%	< 0.001
Low Falls, By Rankin Score						
0		5	9.3%			
1		10	18.5%			
2		12	22.2%			
3		11	20.4%			
4		12	22.2%			
5		4	7.4%			
Low Falls with a Recorded AIS of 3 or more in:*						
External Region (Skin, exc. burns)		0	0.00%	0	0.00%	–
Face		0	0.00%	0	0.00%	–
Head		4	0.18%	239	1.22%	< 0.001
Head† AIS ≥ 3	On anti-platelet on discharge	2	0.10%	104	0.64%	0.001
	On anti-coagulation on discharge	0	0%	14	0.86%	0.24
	On anti-platelet or anti-coagulation on discharge	2	0.09%	114	0.66%	< 0.001
Lower Extremities		3	0.13%	135	0.69%	< 0.001
Neck		0	0.00%	1	0.01%	1.000
Spine		1	0.04%	19	0.10%	0.715
Thorax		0	0.00%	10	0.05%	0.613
Upper Extremities		0	0.00%	2	0.01%	1.000

* AIS: Abbreviated Injury Score; † proportion with denominator as number of persons on relevant anti-thrombotic treatment (i.e. anti-platelet, anticoagulant and either)

Patients with mRS recorded had a mean age of 66.6 years and 58.5% were men. The ethnic composition was reflective of that of the general local population. A large proportion had hypertension (81.5%) and hyperlipidaemia (91.2%), as well as diabetes mellitus (42.1%) or atrial fibrillation (20.3%). Patients who ever smoked represented 41.4% of the sample. By the end of 2015, of those who had a recorded mRS, 54 (2.39%) suffered a low fall while 93 (4.12%) died. The median mRS was 2 (IQR 1–3). Moderate to severe injuries (AIS ≥3) were sustained to the head (*n* = 4), lower extremities (*n* = 3) and spine (*n* = 1).

**Fig. 2:**
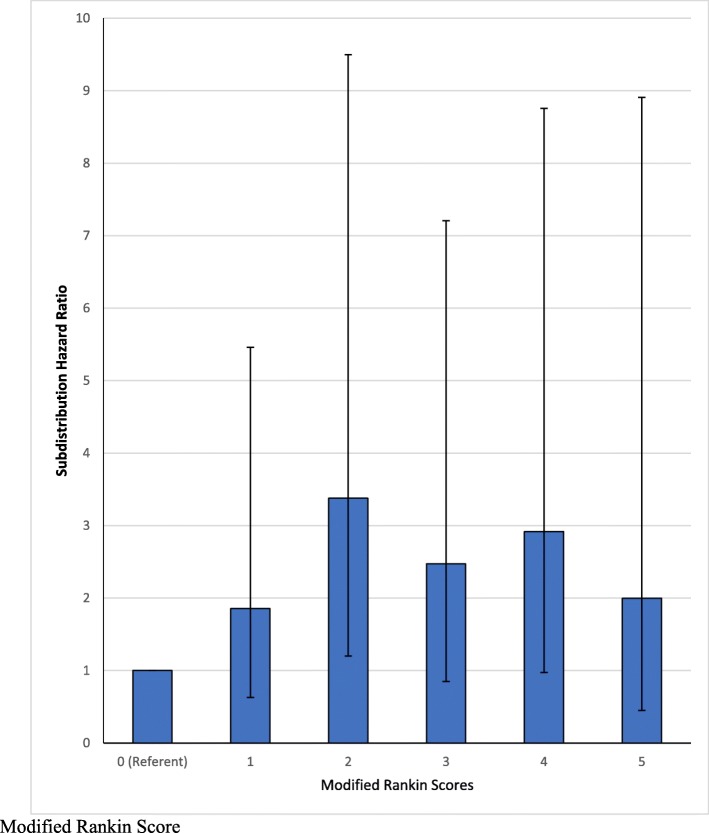
Subdistribution Hazard Ratios of Sustaining a Fall After an Ischaemic Stroke, by Modified Rankin Score

After adjusting for potential confounders and accounting for deaths, the mRS was associated with falls risk and showed an inverse U-shaped relationship (Table 2 and Fig. 2). Compared to patients with a score of 0, the subdistribution hazard ratio (SHR) increased to a maximum of 3.42 (95%CI: 1.21–9.65, *p* = 0.020) for patients with a score of 2. The SHR then declined to 2.45 (95%CI: 0.85–7.12, *p* = 0.098), 2.86 (95%CI: 0.95–8.61, *p* = 0.062) and 1.93 (95% CI 0.44–8.52, *p* = 0.38) for patients with scores of 3, 4 and 5 respectively.

**Table 2:** Competing Risks Analysis Showing Factors Associated with Sustaining a Recorded Fall After Having an Ischemic Stroke

Variables	SHR	95% CI	*p*-value
Final Modified Rankin Score			
0	Referent		
1	1.87	(0.63, 5.50)	0.256
2	3.42	(1.21, 9.65)	0.020
3	2.45	(0.85, 7.12)	0.098
4	2.86	(0.95, 8.61)	0.062
5	1.93	(0.44, 8.52)	0.383
Age (year)	1.01	(0.99, 1.04)	0.366
Blood Sugar Level (mmol/L)	1.00	(0.92, 1.08)	0.912
Haemoglobin at Admission (g/dL)	0.95	(0.83, 1.10)	0.505
Gender			
Female	Referent		
Male	0.40	(0.19, 0.84)	0.015
Ethnicity			
Chinese	Referent		
Malay	0.45	(0.17, 1.18)	0.104
Indian	0.75	(0.22, 2.50)	0.634
Others	1.15	(0.16, 8.41)	0.887
Smoking			
Non-smoker	Referent		
Ex-smoker	2.23	(0.84, 5.93)	0.109
Current-smoker	1.72	(0.79, 3.76)	0.175
Diabetes Mellitus	1.45	(0.78, 2.70)	0.242
Hypertension	1.26	(0.56, 2.82)	0.575
Hyperlipidaemia	1.59	(0.47, 5.33)	0.455
Atrial Fibrillation	0.71	(0.31, 1.62)	0.412
History of TIA or Stroke	1.30	(0.68, 2.49)	0.431
Anti-platelet at discharge	1.30	(0.36, 4.66)	0.687
Anti-coagulant at discharge	1.59	(0.39, 6.41)	0.514
Discharge Home	1.44	(0.77, 2.69)	0.252

Male gender was associated with reduced falls risk (SHR 0.40, 95%CI: 0.19–0.84, *p* = 0.015).

Sensitivity analysis by treating admission haemoglobin levels and blood sugar levels as categorical variables did not alter the associations observed. Antiplatelets and anticoagulants among study patients (patients with mRS recorded) were noted to be associated with greater hazards of falls although this was not statistically significant (antiplatelet SHR 1.30, 95%CI: 0.36–4.66, *p* = 0.69; anticoagulation SHR 1.59, 95%CI: 0.40–6.41, *p* = 0.51). This was further explored with a competing risks analysis for falls performed on all stroke patients (*n* = 24,344 stroke survivors with 2234 falls under complete case analysis) which showed that anticoagulation (SHR1.78, 95%CI 1.48–2.13, *p* < 0.001) and antiplatelet agents (SHR1.50, 95%CI 1.30–1.74, p < 0.001) were associated with greater risk of recorded falls. However, this analysis did not account for functional status as represented by mRS. Analysis of post-fall survival was also performed on this larger sample (*n* = 2234 falls with 559 deaths) which showed that antithrombotic treatment was not associated with worse survival (antiplatelet HR1.05, 95%CI: 0.80–1.37, *p* = 0.73; anticoagulation HR 0.95, 95%CI 068–1.32, *p* = 0.74).

A similar analysis could not be performed on the sample of patients with recorded Rankin score due to no post-fall deaths observed in this group. Compared to patients without mRS (Table 1), those with recorded scores were more likely to be on anticoagulation (11.8% vs 8.3%, *p* < 0.001), be on the stroke pathway of care (93.6% vs 73.2%, *p* < 0.001) and be discharged home (69.3% vs 56.7%, *p* < 0.001). Those with recorded mRS were also less likely to die by 2015 (20.7% vs 4.1%, *p* < 0.001), to sustain low falls (10.2% vs 2.4%, *p* < 0.001) and to sustain severe injuries from falls that had AIS of 3 or more (head injury 0.18% vs 1.22%, *p* < 0.001; lower extremity injury 0.13% vs 0.69%, *p* < 0.001).

## Discussion

We observed an inverse U-shaped relationship between Rankin score and the risk of post-stroke falls. The highest risk was at an mRS of 2. This may be explained by an interplay between exposure to circumstances where falls may occur and one’s physiological susceptibility to falls. Persons with low functional status are less likely to be mobile, are more likely to be physically inactive and hence are exposed to fewer circumstances where they may sustain a fall. On the other hand, persons with higher functional states are more likely to have intact motor and sensory functions for maintaining balance, and hence less likely to fall. Persons with moderate functionality experience a mix of these. They have an impaired ability to maintain balance yet retain some reasonable mobility, and thus attempt to be mobile. This may explain why persons in the middle of the spectrum of physical function are most prone to falls.

This finding corroborates with what has been found in three other studies which showed a similar relationship between function and post-stroke falls [17–19]. Two of these studies are well-powered and utilised cohort study designs [17, 18], one of which used a large registry-based cohort with data on long-term outcomes [17]. In contrast, most other studies demonstrate that worse disability and function are associated with increased falls risk [5, 20, 21]. These studies often dichotomize physical function [21, 22] or analyse functional scores as continuous variables on a linear model [20] which may obscure this observation. Future studies may assess if patterns of falls (e.g. mechanism of fall, location) sustained by patients differ between functional levels. Targeted interventions could be potentially developed based on these and evaluated.

The potential positive association between antithrombotic therapy (anticoagulation and antiplatelets) and falls likely arises from a bias in the detection of falls. Patients on antithrombotic therapy who fall are managed with greater caution due to greater risks of haemorrhage and are more likely to undergo brain scans and receive inpatient care [23, 24]. Also, these patients may be more likely to present for medical attention for falls due to precautions advised in case of trauma while on antithrombotic therapy. We are further reassured that neither agents were associated with worse post-fall survival in the sensitivity analysis. Of note, only 4 (0.18%) of the patients with modified Rankin score sustained a head injury with AIS score of 3 or more during the study period, and of all the ischemic stroke survivors in the registry, only 243 (1.1%) sustained a head injury with an AIS score of 3 or more. Concerns that anti-coagulation may lead to major intracranial haemorrhage after a fall are allayed by the low incidence of serious head injuries and the no-worse survival profile.

### Limitations and strengths

The injurious fall rate in our study is 2.4%, much lower than the proportion of all fallers among community stroke survivors observed in other studies ranging between 23.0 and 55.0% [5] in the literature. This is because the NTR captures only falls leading to emergency department presentations, capturing significant incidents but is not representative of all falls. In contrast, most studies use self-reported fall outcomes either through interviews or diaries [5, 25], which would detect minor falls not picked up by the NTR. In this respect, this study complements the literature on prospective falls, by focusing on falls serious enough to present to hospital. However, it also carries the limitation of omitting near-miss falls or seemingly minor falls, events that are known to have implications for frailty and future falls.

In the multivariable regression model, 15 individual factors were included for being established risk factors for falls. While this may risk over-fitting with the additional variables, the explanatory model maintains the inverted U-shaped trend (in relation to mRS) observed in the univariate analysis (Table 1) and confirms that the trend is not due to confounding.

A low proportion of all stroke patients within the registry had mRS recorded (10.3%). There was a significant baseline difference in the history of TIA or stroke (14.4% vs 20.3%), a small but statistically significant difference in age (66.6 years vs 67.7 years), but no significant differences in haemoglobin levels at admission and cardiovascular co-morbidities (diabetes mellitus, hypertension, hyperlipidaemia and atrial fibrillation).

Of note, the patients with recorded mRS included in our analysis were different from those without mRS in several ways. Patients in our study, who had a complete mRS, were more likely to be on a stroke pathway (93.6% vs 73.2%) than those that were excluded, and more likely to be discharged home. They also had a lower mortality (4.1% vs 20.7%), and lower risks of falls (2.4% vs 10.2%), suggesting that there were uncaptured baseline differences in other co-morbidities and functional status that contributed to differences in outcome.

These differences could explain why, at discharge, they were more likely to receive antiplatelet agents (85.1% vs 82.9%) or oral anticoagulation (11.8% vs 8.3%) than the patients missing mRS. Furthermore, patients missing mRS might include patients assessed by clinicians to have higher fall risk or lower mortality benefit, patients with poor compliance to anticoagulation follow-up and patients who might also not be compliant to rehabilitation follow-up where the mRS would be scored. Another explanation is that patients missing mRS likely included patients with subclinical stroke diagnosed incidentally when presenting for another medical problem, or those presenting with multiple medical issues, hence accounting for the much lower proportion treated in stroke units. Hence, our study findings may be generalizable primarily to patients presenting with acute stroke as the main presenting complaint, and less generalizable to patients where stroke is not their primary presenting complaint, as they were more likely to be missing mRS in our registry and excluded from our study.

Another potential bias comes from the timing of the score. As scoring of functional status is commonly done in rehabilitation [26], the sample may select for persons who are deemed more appropriate for rehabilitation and may reflect better health and prognosis. Finally, as with all registry-based studies, we were only able to include risk factors in our model that were mandated in the registries used.

The strengths are that we used nation-wide registry data involving multiple centres with a large number of patients, with data from a 5-year period.

## Conclusions

An inverse U-shaped relationship between functional status and falls risk was observed. This is consistent with the interplay between decreasing mobility (hence opportunities of falling) and increasing susceptibility to falls. Across mRS scores, patients with a score of 2 have the highest risk of falls and are important candidates for preventive intervention.

## Data Availability

The data that support the findings of this study are available from the National Registry of Diseases Office in Singapore, but restrictions apply to the availability of these data, which were used under license for the current study, and so are not publicly available. Data are however available from the authors upon reasonable request and with permission of the National Registry of Diseases Office, Singapore.

## Abbreviations

AIS: Abbreviated Injury Score
HR: Hazard ratio
mRS: Modified Rankin scale
NRDO: National Registry of Diseases Office
NSR: National Stroke Registry
NTR: National Trauma Registry
SHR: Sub-distribution hazard ratio
